# High-Fat Diet Alters Circadian Rhythms in Mammary Glands of Pubertal Mice

**DOI:** 10.3389/fendo.2020.00349

**Published:** 2020-06-18

**Authors:** Sneha Sundaram, LuAnn K. Johnson, Lin Yan

**Affiliations:** U.S. Department of Agriculture, Agricultural Research Service, Grand Forks Human Nutrition Research Center, Grand Forks, ND, United States

**Keywords:** circadian rhythm, mammary glands, puberty, diet, mice

## Abstract

Childhood obesity in girls is associated with early puberty and menarche. Breast tissue exhibits circadian rhythms. These rhythms may be altered by environmental factors. We hypothesized that a high-fat diet (HFD) disrupts circadian rhythms in pubertal mammary glands. Weanling female C57BL/6 mice were fed the standard AIN93G diet or a HFD (providing 16% or 45% of energy from soybean oil) for 3 weeks. Mammary glands were harvested from 6-week-old mice every 4 h on Zeitgeber time over a 48-h period; rhythmic expressions of circadian genes and genes encoding estrogen receptor and progesterone receptor were analyzed by using the Cosinor model. HFD, compared to AIN93G diet, altered diurnal oscillations of circadian genes in pubertal mammary glands. These included changes in amplitude of *Per2, Cry1* (reduced), *Clock, Rev-erb*α, and *Per1* (elevated), a delay in acrophase (the hour at which the rhythm peaks) of *Bmal1* by 2.2 h, and changes in mesor (the mean of the rhythm from peak to trough) of *Bmal1, Per2, Cry1* (reduced), *Rev-reb*α, and *Per1* (elevated). Furthermore, HFD altered diurnal expression of estrogen receptor and progesterone receptor at both mRNA and protein levels. These findings indicate that HFD alters circadian regulation in pubertal mammary glands, which may contribute to the disturbance of hormonal homeostasis and lead to early development and growth of mammary glands in pubertal mice.

## Introduction

The prevalence of overweight and obesity in children has increased substantially worldwide (1). Childhood obesity can have long lasting health impacts. It is a risk factor for chronic diseases including breast cancer. Obese girls exhibit an earlier onset of puberty (2, 3) and breast development (4, 5) with the elevation of ovarian hormones. The earlier age of menarche is associated with an increased risk of breast cancer in adult women (6, 7). Breast cancer remains a leading cause of death for women worldwide (8).

Childhood obesity occurs with lifestyle changes in the modern world (e.g., erratic eating and sedentary behavior). These lifestyle choices, as environmental factors, disrupt diurnal patterns of circadian rhythms and contribute to metabolic dysfunction and obesity (9–11). All biological systems exhibit circadian rhythms that cycle approximately every 24 h from gene expression to physiological functions (e.g., fasting vs. eating) (12–14). The central clock is located in the hypothalamus, and peripheral clocks are in all other organs. The synchrony between the central and peripheral clocks regulates metabolism to set temporal rhythms in homeostatic regulation for health and well-being (12–14). The central clock is controlled by light input via the retina and passes these signals onto peripheral clocks.

Peripheral organs, including mammary glands, have their own intrinsic self-sustained circadian oscillations, which are sensitive to environmental changes (e.g., erratic eating) but depend on the central clock for synchronization (15, 16). At the molecular level, both central and peripheral clocks consist of interconnected transcription-translation feedback loops that regulate daily circadian oscillations. Activation of circadian locomotor output cycles kaput/aryl hydrocarbon receptor nuclear translocator-like protein 1 (*Clock/Bmal1*) heterodimers occurs during the rest phase (the light phase for nocturnal animals) and leads to the transcription of many clock-controlled genes, including period (*Per)* and cryptochrome (*Cry*). The *Per*/*Cry* dimers reach peak expression during the late rest phase or early active phase (the dark phase for nocturnal animals) and interact with *Clock/Bmal1* to repress their transcription via a negative-feedback loop. This, in turn, leads to the start of a new cycle of *Clock/Bmal1* transcription in the rest phase. *Clock/Bmal1* dimers also activate the expression of nuclear receptor subfamily 1 group D member 1 (*Rev-erb*) that leads to the repression of *Clock/Bmal1*.

Consumption of a high-fat diet, as an environmental factor, disrupts the meticulously coordinated circadian system and results in metabolic dysfunction (9, 17). For example, a high-fat diet alters the eating pattern of mice with more food intake during the light phase (10), leading to phase-advance of *Per2* in liver (11) and blunting the rhythmicity of *Clock, Bmal1*, and *Per2* in liver (18, 19) and adipose tissue (18). The dampened expression of circadian genes occurs concurrently with the increases in proinflammatory cytokines and body adiposity in diet-induced obese mice (10).

Mammary glands are unique because development mainly occurs post-embryonically during puberty. The ovarian steroid hormones are involved in mammary development. Estrogen directly acts on mammary glands; estrogen receptor 1 regulates ductal morphogenesis (20, 21) and estrogen receptor 2 facilitates terminal differentiation (22). Progesterone stimulates mammary epithelial DNA synthesis and alveolar development through progesterone receptor (23, 24). Disruption of the receptor expression alters mammary development. For example, mammary glands from estrogen receptor 1 knockout mice do not undergo ductal morphogenesis (25) while those from progesterone receptor knockout mice are arrested at the simple ductal stage (24). There is growing evidence that circadian regulation plays a role in breast development and growth (26, 27).

Feeding weanling mice a high-fat diet disrupts daily oscillations of energy expenditure (28), increases body adiposity (28), and enhances mammary tumorigenesis in a mouse mammary tumor virus-polyomavirus middle T-antigen (MMTV-PyMT) model of breast cancer (29). Time-restricted feeding of weanling mice during the dark phase reduces the high-fat diet-enhanced mammary tumorigenesis (30). Time-restricted feeding is an effective tool in circadian research that restores daily rhythms in rodent models, evidenced by the restored diurnal oscillations of circadian genes (17) and energy metabolism (28).

The above findings support the concept that disruption of circadian rhythms at early stages of mammary development contributes to diet-enhanced mammary tumorigenesis. However, there is a lack of knowledge of the degree to which diet alters diurnal expression of circadian genes in mammary glands of pubertal mice. We hypothesized that a high-fat diet disrupts diurnal expression of circadian genes in pubertal mammary glands. This study tested this hypothesis by comparing differences in diurnal expressions of circadian genes and genes encoding estrogen receptor and progesterone receptor in mammary glands from pubertal mice fed the AIN93G diet or a high-fat diet.

## Materials and Methods

### Animals and Diets

Weanling 3-week-old female C57BL/6 mice (Envigo, Madison, WI, USA) were housed, three per cage, in a pathogen-free room with a 12:12-h light/dark cycle and a temperature of 22 ± 1°C. The light intensity of the light phase was 32 lux. The standard AIN93G diet (31) and a modified AIN93G diet containing 45% of energy from soybean oil (hereafter referred to as the high-fat diet, HFD) were used in this study (Table 1). HFD was adjusted to provide equal amounts of vitamins and minerals on a per caloric basis to AIN93G diet (Table 1). Gross energy of each diet (Table 1) was quantified by using an oxygen bomb calorimeter (Model 6,200, Parr Instrument, Moline, IL, USA). Both diets (powder form) were kept at −20°C. Fresh diets were provided to mice every other day.

**Table 1:** Composition of diets.

	**AIN93G**	**High-Fat**
Ingredient	g/kg	g/kg
Corn Starch	397.5	42.5
Casein	200	239.4
Dextrin	132	239.4
Sucrose	100	119.7
Soybean oil	70	239.4
Cellulose	50	59.8
AIN93 mineral mix	35	41.9
AIN93 vitamin mix	10	12
L-Cystine	3	3.6
Choline bitartrate	2.5	3
*t*-Butylhydroquinone	0.014	0.017
Total	1,000	1,000
Energy	%	%
Protein	20	20
Fat	16	45
carbohydrate	64	35
Analyzed gross energy kcal/g^a^	4.3 ± 0.1	5.2 ± 0.1

a *values are means ± SEM of three samples analyzed from each diet*.

### Experimental Design

After acclimation on AIN93G diet for 2 days, mice were randomly assigned to two groups of 70 each and fed AIN93G or HFD for 3 weeks. Mice had free access to their diets and deionized water and were weighed weekly. To avoid potential circadian disruption by housing mice in metabolic cages and to avoid exposure to magnetic resonance, food intake and body composition assessments were not performed. At the end of the study, five mice from each group were euthanized by an intra-peritoneal injection of a mixture of ketamine and xylazine at Zeitgeber time 0 (light on), 4, 8, 12 (light off), 16, 20, and 24 over a period of 48 h. Mammary glands of each mouse from thoracic, abdominal, and inguinal locations were collected and pooled, snap-frozen in liquid nitrogen, and stored at −80°C.

### RNA Isolation and Real-Time Quantitative PCR

Total RNA was isolated from mammary glands by using the QIAzol Lysis reagent with DNAse treatment (RNeasy Lipid Tissue Mini Kit mRNA, Qiagen, Germantown, MD, USA). The quantity and quality of purified RNA were evaluated by using the NanoDrop 8000 Spectrophotometer (Thermo Scientific, Wilmington, DE, USA). cDNA was synthesized by using the high capacity cDNA reverse transcription kit (Applied Biosystems, Waltham, MA, USA). Real-time qPCR of *Clock (*Mm00455950_m1*), Bmal1 (*Mm00500223_m1*), Per1 (*Mm00501813_m1*)* and *Per2* (Mm00478099_m1*), Cry1 (*Mm00514392_m1*), Rev-erb*α *(*Mm00520708_m1*), estrogen receptors 1 (Esr1)* (Mm00433149_m1*)* and *2 (Esr2) (*Mm00599821_m1*)*, and *progesterone receptor* (*Pgr*) (Mm00435628_m1) were analyzed and normalized to the 18s rRNA (Mm03928990_g1) by using the TaqMan predesigned gene-specific probes on the ABI QuantStudio 12K-Flex Real-time PCR system (Applied Biosystems). The relative changes in gene expression were calculated by using the 2^−ΔΔCT^ method (32).

### Quantification of Estradiol, Progesterone, and Their Receptors in Mammary Glands

Total protein was extracted from mammary glands by using the radioimmunoprecipitation assay buffer (R2078, Sigma, St Louis, MO, USA) with protease and phosphatase inhibitors (33, 34). Protein concentrations of mammary extracts were quantified by using the bicinchoninic acid protein assay (Thermo Scientific). Sandwich enzyme-linked immunosorbent assays were performed to quantify estradiol (Biovision, Milpitas, CA, USA), progesterone (MyBioSource, San Diego, CA, USA), estrogen receptor 1 and 2, and progesterone receptor (LifeSpan BioSciences, Seattle, WA, USA) in mammary glands. Samples were read within the linear range of the assay. The accuracy of the analysis was confirmed by the controls provided in each kit.

### Statistical Analyses

Weekly body weight differences between the two dietary groups were analyzed by using a repeated measures analysis of variance followed by Tukey contrasts at each week. Student's *t*-test was performed to compare differences in concentrations of estradiol, progesterone, and their receptors in mammary glands between the two groups. The rhythmic nature of the gene and protein expression was analyzed by using the Cosinor model. Specifically, the model was y = mesor + amplitude x Cos [2π/24 × (t—acrophase)], where mesor (midline estimating statistic of rhythm) = the mean of the oscillations, amplitude = the distance between the mesor and the peak of the oscillations, acrophase = the hour at which the maximum value of the cosine wave (the peak of the rhythm) occurs, and t = time in hours. The period of the oscillations was assumed to be 24 h. Nested non-linear models were used to determine if separate model parameters (mesor, amplitude, and acrophase) were needed for each dietary group or if common parameters were sufficient. In these analyses, parameters were estimated directly for the AIN93G group, while the parameters for the HFD group were expressed as offsets from the corresponding AIN93G parameters. If the 95% confidence interval (95% CI) of the offset did not cross 0, the parameters were considered different between the AIN93G and HFD groups (*p* < 0.05) (35). All models were fit by using the NLIN procedure in SAS software version 9.4 (SAS Institute, Inc., Cary, NC, USA).

## Results

### Body Weight

**Figure 1:**
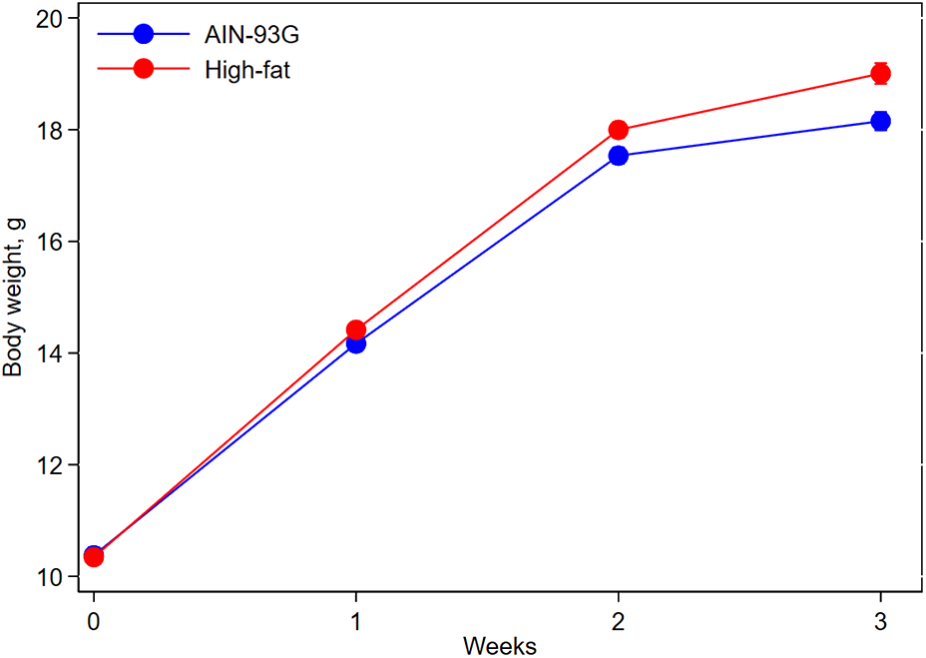
Body weight of pubertal mice fed AIN93G or high-fat diet. Mice fed high-fat diet were slightly heavier than mice fed AIN93G diet. The difference was significant at week 2 (*p* < 0.05) and week 3 (*p* < 0.01) after the high-fat diet (*n* = 70 per group).

Mice fed HFD were slightly heavier than mice fed AIN93G diet; the difference was significant 2 weeks after the initiation of HFD (*p* < 0.05) and continued throughout the study (Figure 1). At the end of the study, body weight of mice fed AIN93G diet was 18.2 ± 0.2 g and that of mice fed HFD was 19.0 ± 0.2 g (*n* = 70 per group).

### Expression of Circadian Genes in Mammary Glands

**Figure 2:**
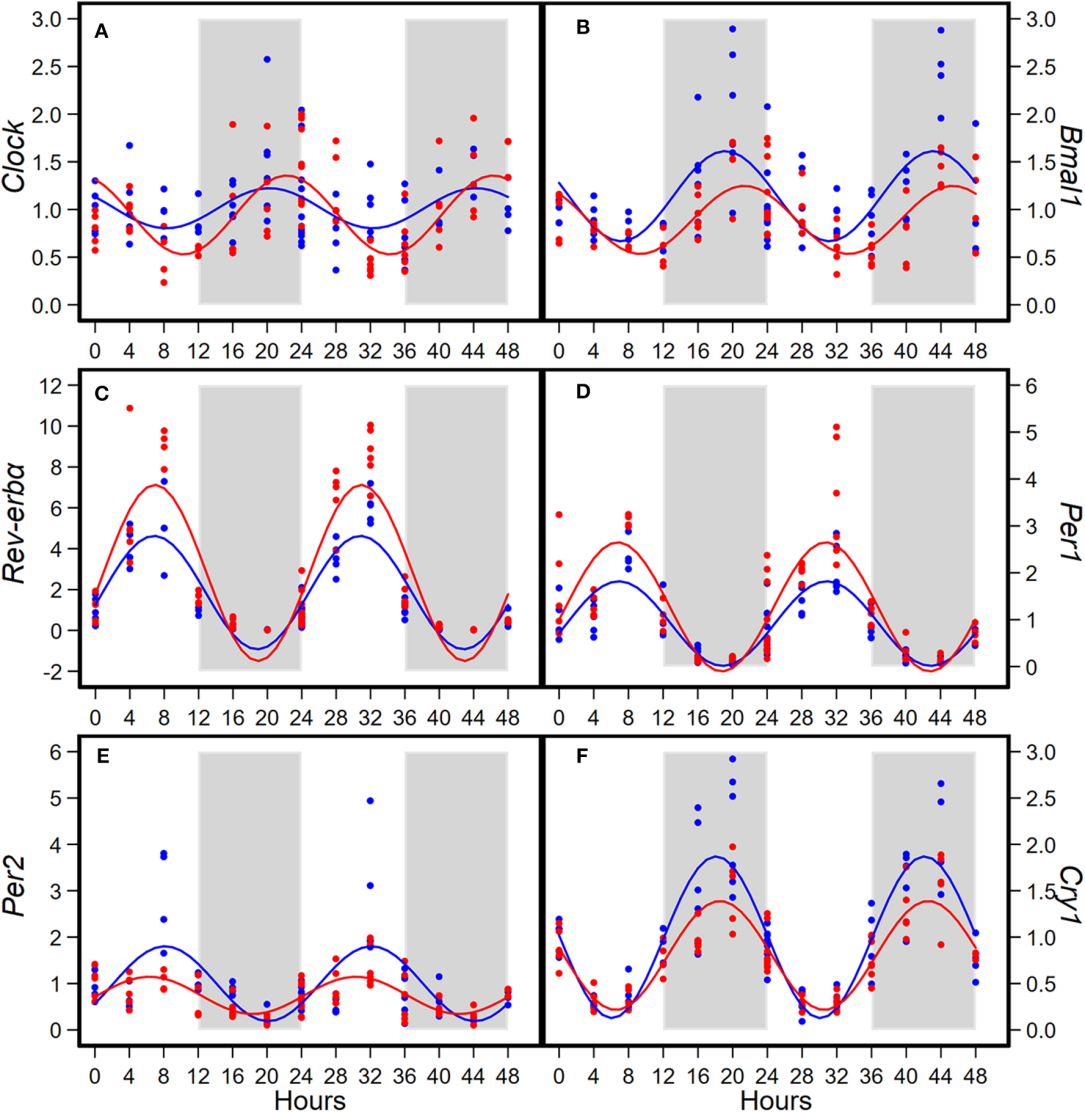
Diurnal expressions of *Clock* **(A)**, *Bmal1* **(B)**, *Rev-erb*α **(C)**, *Per1* **(D)**, *Per2* **(E)**, and *Cry1* **(F)** in mammary glands from pubertal mice fed AIN93G or high-fat diet (*n* = 5 per time point per group). Open background: light phase; gray background: dark phase; blue circles and line: AIN93G diet; red circles and line: high-fat diet. The rhythm curves were generated by using the Cosinor model y = mesor + amplitude x Cos [2π/24 × (t—acrophase)].

Consumption of HFD altered diurnal oscillations of circadian genes in mammary glands (Figure 2). Compared to AIN93G diet, HFD induced >30% reduction in amplitude of *Per2* and *Cry1* and >50% elevation in amplitude of *Clock, Rev-erb*α, and *Per1* (Figure 2, Table 2). HFD also delayed the acrophase of *Bmal1* by 2.2 h (Figure 2B, Table 2). There was no difference in acrophase between the two groups for the other five circadian genes analyzed (Table 2). Compared to AIN93G diet, HFD resulted in >20% reduction in mesor of *Bmal1, Per2*, and *Cry1* and >30% elevation in mesor of *Rev-erb*α and *Per1* (Figure 2, Table 2).

**Table 2:** Estimated values obtained from the Cosinor model of fold changes in expression of circadian genes in mammary glands from pubertal mice fed AIN93G or high-fat diet.

**mRNA**	**Amplitude**			***p***
	**AIN93G^a^**	**High-Fat^a^**	**Difference (95% CI)^b^**	
*Clock*	0.21 ± 0.07	0.41 ± 0.06	0.20 (0.02 to 0.38)	0.03
*Bmal1*	0.47 ± 0.07	0.36 ± 0.07	−0.12 (−0.31 to 0.08)	0.24
*Rev-erbα*	2.77 ± 0.25	4.31 ± 0.25	1.54 (0.84 to 2.24)	<0.01
*Per1*	0.90 ± 0.11	1.38 ± 0.11	0.47 (0.18 to 0.77)	<0.01
*Per2*	0.81 ± 0.10	0.40 ± 0.10	−0.41 (−0.68 to 0.13)	<0.01
*Cry1*	0.87 ± 0.06	0.59 ± 0.06	−0.28 (−0.44 to −0.13)	<0.01
**mRNA**	**Acrophase (Hours)**			***p***
	**AIN93G**^a^	**High-Fat**^a^	**Difference (95% CI)**^b^	
*Clock*	20.27 ± 1.13	22.28 ± 0.61	2.00 (−0.53 to 4.54)	0.12
*Bmal1*	19.15 ± 0.52	21.35 ± 0.73	2.21 (0.44 to 3.97)	0.02
*Rev-erbα*	6.83 ± 0.31	6.93 ± 0.20	0.10 (−0.63 to 0.82)	0.79
*Per1*	6.89 ± 0.40	6.77 ± 0.26	−0.11 (−1.07 to 0.84)	0.82
*Per2*	8.14 ± 0.43	6.33 ± 0.85	−1.80 (−3.68 to 0.08)	0.06
*Cry1*	18.09 ± 0.22	18.54 ± 0.33	0.44 (−0.34 to 1.22)	0.26
**mRNA**	**Midline Estimating Statistic of Rhythm (mesor)**			***p***
	**AIN93G**^a^	**High-Fat**^a^	**Difference (95% CI)**^b^	
*Clock*	1.01 ± 0.05	0.94 ± 0.05	−0.07 (−0.20 to 0.06)	0.27
*Bmal1*	1.14 ± 0.05	0.89 ± 0.05	−0.25 (−0.38 to −0.12)	<0.01
*Rev-erbα*	1.85 ± 0.17	2.81 ± 0.17	0.96 (0.49 to 1.43)	<0.01
*Per1*	0.92 ± 0.07	1.27 ± 0.07	0.36 (0.16 to 0.56)	<0.01
*Per2*	1.00 ± 0.07	0.74 ± 0.07	−0.25 (−0.44 to −0.06)	0.01
*Cry1*	1.00 ± 0.04	0.80 ± 0.04	−0.20 (−0.30 to −0.09)	<0.01

a *estimates ± standard error (SE) for the AIN93G and the high-fat diet groups*.

b *estimated difference and its 95% confidence interval (95% CI) between the AIN93G and the high-fat diet groups*.

### Expression of Estrogen Receptor and Progesterone Receptor Genes in Mammary Glands

**Figure 3:**
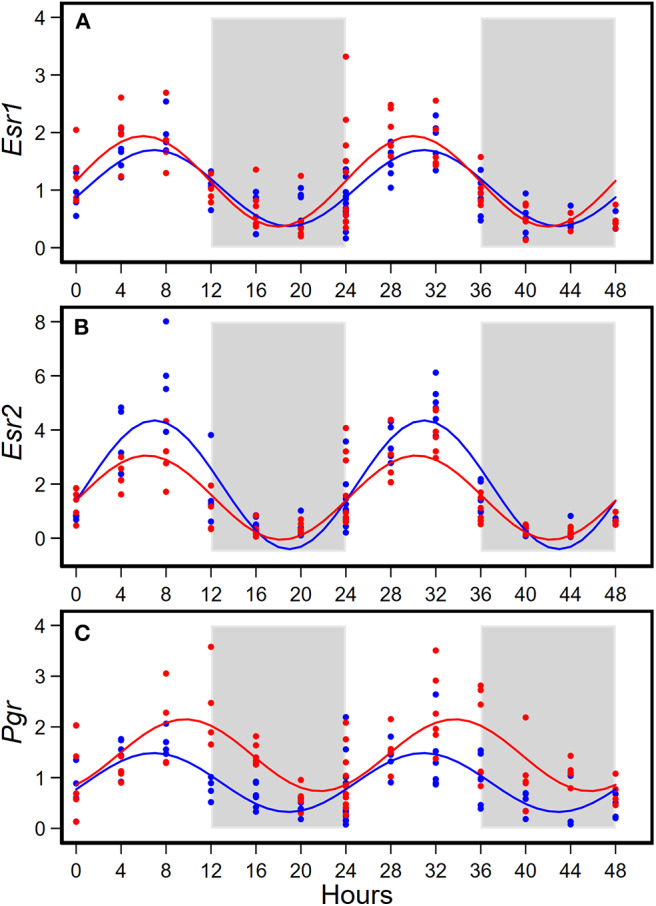
Diurnal expressions of genes encoding *estrogen receptor 1* (*Esr1*, **A**), *estrogen receptor 2* (*Esr2*, **B**), and *progesterone receptor* (*Pgr*, **C**) in mammary glands from pubertal mice fed AIN93G or high-fat diet (*n* = 5 per time point per group). Open background: light phase; gray background: dark phase; blue circles and line: AIN93G diet; red circles and line: high-fat diet. The rhythm curves were generated by using the Cosinor model y = mesor + amplitude x Cos [2π/24 × (t—acrophase)].

HFD altered diurnal expression of genes encoding estrogen receptor and progesterone receptor in mammary glands (Figure 3, Table 3). There were no significant differences in diurnal oscillations of *Esr1* between the two groups, regarding amplitude, acrophase, and mesor (Figure 3A, Table 3). HFD, compared to AIN93G diet, resulted in a >30% reduction in amplitude and a 20% reduction in mesor of *Esr2* (Figure 3B, Table 3). HFD also delayed acrophase of *Pgr* by 2.8 h and elevated the mesor of *Pgr* by 60% (Figure 3C, Table 3).

**Table 3:** Estimated values obtained from the Cosinor model of fold changes in the expression of genes encoding estrogen receptors and progesterone receptor in mammary glands from pubertal mice fed AIN93G or high-fat diet.

**mRNA**	**Amplitude**			***p***
	**AIN93G^a^**	**High-Fat^a^**	**Difference (95% CI)^b^**	
*Esr1*	0.66 ± 0.08	0.79 ± 0.08	0.13 (−0.10 to 0.35)	0.28
*Esr2*	2.38 ± 0.17	1.56 ± 0.17	−0.82 (−1.29 to −0.36)	<0.01
*Pgr*	0.58 ± 0.10	0.71 ± 0.09	0.13 (−0.13 to 0.39)	0.33
mRNA	Acrophase (Hours)			*p*
	AIN93G^a^	High-Fat^a^	Difference (95% CI)^b^	
*Esr1*	6.93 ± 0.42	5.96 ± 0.35	−0.97 (−2.05 to 0.11)	0.08
*Esr2*	6.96 ± 0.24	6.27 ± 0.36	−0.70 (−1.55 to 0.16)	0.11
*Pgr*	6.90 ± 0.56	9.69 ± 0.50	2.79 (1.31 to 4.28)	<0.01
mRNA	Midline estimating statistic of rhythm (mesor)			*p*
	AIN93G^a^	High-Fat^a^	Difference (95% CI)^b^	
*Esr1*	1.04 ± 0.05	1.15 ± 0.05	0.12 (−0.04 to 0.27)	0.13
*Esr2*	1.97 ± 0.11	1.50 ± 0.11	−0.47 (−0.79 to −0.16)	<0.01
*Pgr*	0.90 ± 0.06	1.44 ± 0.06	0.54 (0.36 to 0.72)	<0.01

a *estimates ± standard error (SE) for the AIN93G and the high-fat diet groups*.

b *estimated difference and its 95% confidence interval (95% CI) between the AIN93G and the high-fat diet groups*.

### Concentrations of Estrogen, Progesterone, and Their Receptors in Mammary Glands

**Figure 4:**
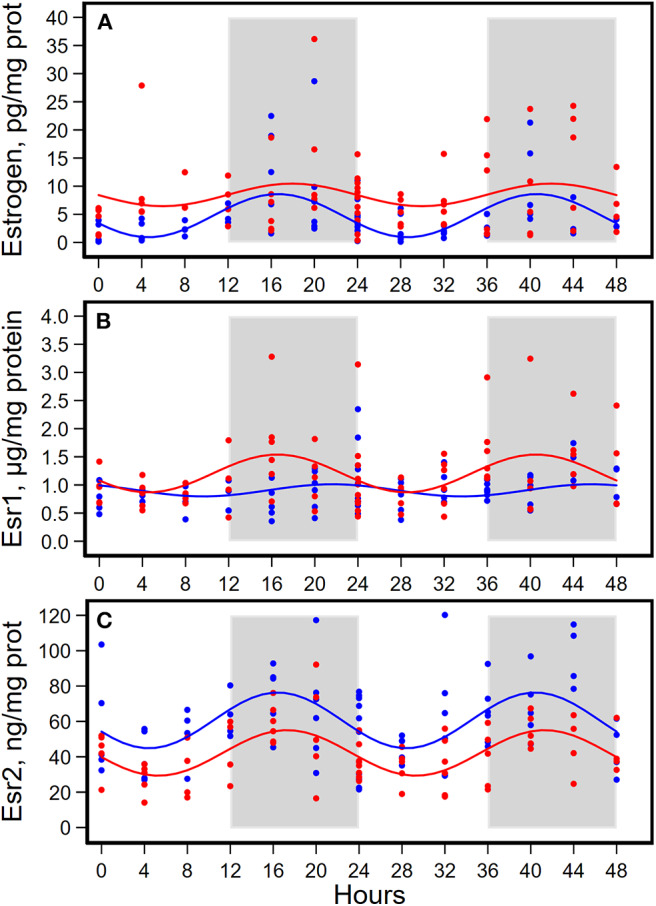
Diurnal expressions of concentrations of estrogen **(A)**, estrogen receptor 1 (Esr1, **B**), and estrogen receptor 2 (Esr2, **C**) in mammary glands from pubertal mice fed AIN93G or high-fat diet (*n* = 5 per time point per group). Open background: light phase; gray background: dark phase; blue circles and line: AIN93G diet; red circles and line: high-fat diet. The rhythm curves were generated by using the Cosinor model y = mesor + amplitude x Cos [2π/24 × (t—acrophase)].

**Figure 5:**
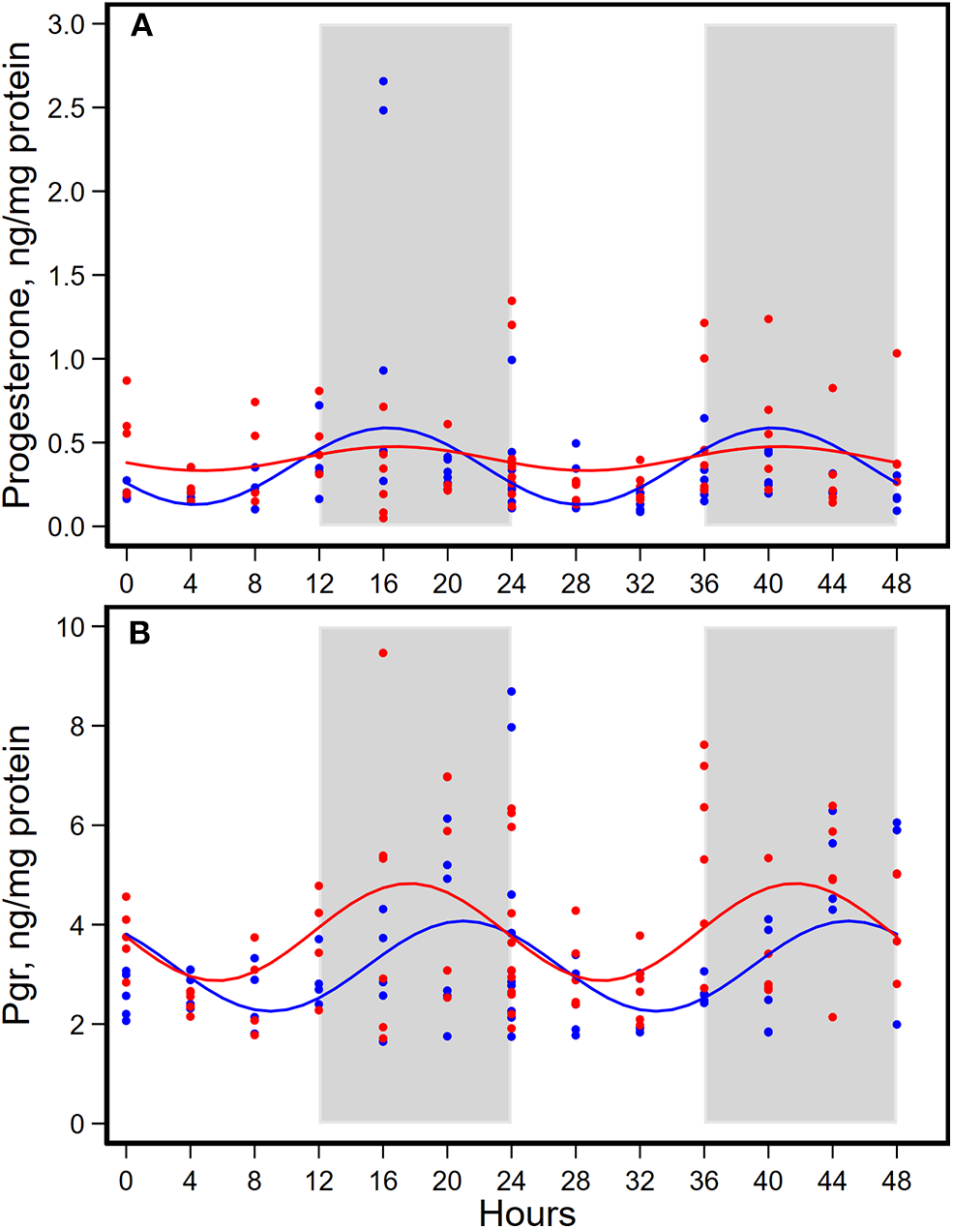
Diurnal expressions of concentrations of progesterone **(A)** and progesterone receptor (Pgr, **B**) in mammary glands from pubertal mice fed AIN93G or high-fat diet (*n* = 5 per time point per group). Open background: light phase; gray background: dark phase; blue circles and line: AIN93G diet; red circles and line: high-fat diet. The rhythm curves were generated by using the Cosinor model y = mesor + amplitude x Cos [2π/24 × (t—acrophase)].

HFD altered diurnal oscillations of concentrations of estrogen, progesterone, and their receptors in mammary glands (Figures 4, 5, Table 4).

**Table 4:** Estimated values from the Cosinor model of concentrations of estrogen, progesterone, and their receptors in mammary glands from pubertal mice fed AIN93G or high-fat diet.

	**Amplitude**			
	**AIN93G^a^**	**High-Fat^a^**	**Difference (95% CI)^b^**	***p***
Estrogen, pg/mg	3.84 ± 1.06	2.01 ± 1.04	−1.83 (−4.78 to 1.12)	0.22
Esr1, μg/mg	0.11 ± 0.08	0.34 ± 0.09	0.23 (−0.02 to 0.47)	0.07
Esr2, ng/mg	15.77 ± 3.29	12.88 ± 3.32	−2.89 (−12.13 to 6.36)	0.54
Progesterone, ng/mg	0.23 ± 0.06	0.07 ± 0.06	−0.16 (−0.33 to 0.02)	0.08
Pgr, ng/mg	0.91 ± 0.25	0.98 ± 0.26	0.07 (−0.65 to 0.79)	0.85
	**Acrophase (Hours)**			
	**AIN93G**^a^	**High-Fat**^a^	**Difference (95% CI)**^b^	***p***
Estrogen	16.63 ± 0.99	17.94 ± 1.76	1.32 (−2.68 to 5.31)	0.52
Esr1	21.63 ± 3.16	16.53 ± 0.95	−5.10 (−11.62 to 1.42)	0.12
Esr2	16.42 ± 0.73	17.30 ± 0.90	0.87 (−1.43 to 3.17)	0.46
Progesterone	16.26 ± 0.97	16.68 ± 3.06	0.43 (−5.93 to 6.78)	0.89
Pgr	21.00 ± 1.06	17.63 ± 0.92	−3.37 (−6.15 to −0.58)	<0.05
	**Midline estimating statistic of rhythm (mesor)**			
	**AIN93G**^a^	**High-Fat**^a^	**Difference (95% CI)**^b^	***p***
Estrogen, pg/mg	4.77 ± 0.73	8.43 ± 0.70	3.67 (1.67 to 5.66)	<0.01
Esr1, μg/mg	0.90 ± 0.06	1.21 ± 0.06	0.30 (0.13 to 0.47)	<0.01
Esr2, ng/mg	60.60 ± 2.23	42.19 ± 2.26	−18.40 (−24.68 to 12.12)	<0.01
Progesterone, ng/mg	0.36 ± 0.04	0.40 ± 0.04	0.05 (−0.07 to 0.16)	0.45
Pgr, ng/mg	3.17 ± 0.18	3.85 ± 0.18	0.68 (0.19 to 1.18)	<0.01

a *estimates ± standard error (SE) for the AIN93G and the high-fat diet groups*.

b *estimated difference and its 95% confidence interval (95% CI) between the AIN93G and the high-fat diet groups*.

HFD elevated mesor of estrogen by 70%, but it did not result in significant changes in amplitude and acrophase of estrogen (Figure 4A, Table 4). HFD resulted in a 30% elevation in Esr1 mesor (Figure 4B, Table 4) and a 30% reduction in Esr2 mesor (Figure 4C, Table 4). Differences in mesor of estrogen and its receptors between the two groups were reflected by changes in their respective concentrations in mammary glands. HFD elevated estrogen by 90%, Esr1 by 30%, and reduced Esr2 by 30% (Table 5).

**Table 5:** Concentrations of estrogen, progesterone, and their receptors in mammary glands from pubertal mice fed AIN93G or high-fat diet.

	**AIN93G**	**High-Fat**	***p***
Estrogen, pg/mg	4.50 ± 0.59	8.45 ± 0.83	<0.01
Esr1, μg/mg	0.92 ± 0.04	1.19 ± 0.08	<0.01
Esr2, ng/mg	59.69 ± 2.86	41.88 ± 1.97	<0.01
Progesterone, ng/mg	0.34 ± 0.05	0.40 ± 0.04	0.35
Pgr, ng/mg	3.26 ± 0.18	3.85 ± 0.20	0.03

*N = 70 each for the AIN93G and the high-fat diet groups*.

HFD did not alter the expression of progesterone in amplitude, acrophase, or mesor (Figure 5A, Table 4). HFD advanced acrophase of Pgr by 3.4 h and elevated mesor of Pgr by 20% (Figure 5B, Table 4). HFD elevated Pgr by 20%, but there was no significant difference in the concentration of progesterone between the two dietary groups (Table 5).

## Discussion

The present study showed diurnal expression of circadian genes in mammary glands of pubertal mice. HFD altered the rhythmic expression of circadian genes in mammary glands, evidenced by changes in amplitude, acrophase, or mesor of the rhythmic oscillations. These findings support our hypothesis that HFD disrupts diurnal expression of circadian genes in pubertal mammary glands.

Alterations in diurnal expression of circadian genes by HFD indicate that a high-fat consumption can disrupt the circadian clock in pubertal mammary glands. It has been suggested that the circadian clock is involved in cellular pathways critical for cell division and breast homeostasis. For example, elevated *Per2* expression is correlated positively with *cMyc* and *Cyclin D1* levels, whereas expression of *Per1* and *Bmal1* is correlated with β*-casein* in HC-11 mouse mammary epithelial cells and in developing mammary glands of growing C57BL/6J mice (26). Other studies have found that *Per2* mutant mice exhibit deregulated expression of *cMyc* and *Cyclin D1* (36) and that overexpression of *Per2* reduces cell proliferation and induces apoptosis in EMT6 mammary epithelial cells (37). It also has been shown that the rhythmic expression of circadian genes in mammary glands varies at different stages of breast development (26, 38). While the aim of the present study was not to elucidate the role of circadian regulation in mammary gland development of pubertal mice, it suggests that alterations in diurnal circadian gene expression by a high-fat diet may disturb mammary development and growth at the pubertal stage.

Circadian genes have alternative biological functions, independent from their circadian functions, in mammary development. For example, *Per2* expression is high in virgin mammary glands and *Per1* expression is the highest in lactating mammary glands (26). The findings that *Per2* deficient mice exhibit fewer bifurcations and a lack of distal migration of the ducts is evidence that *Per2* participates in mammary branching morphogenesis (39). Providing further evidence for this, *Clock* mutant mice showed a defect in lactation (40, 41). We found that HFD reduced mesor of *Per2* and elevated that of *Per1* in pubertal mammary glands. These findings suggest the possibility that HFD may disrupt the alternative biological functions of circadian genes, through which it may disturb the normal mammary development and growth in pubertal mice.

In the present study, pubertal mammary glands exhibited diurnal expression of estrogen, progesterone, and their receptors at both mRNA and protein levels. It has been proposed that steroid hormone signaling is interconnected with circadian signaling and that the circadian clock regulates steroid hormones and their receptors. *Per2* deficient mice exhibit increased numbers of Esr1-positive and Pgr-positive cells in mammary glands (39). *In vitro* studies showed that *Per2* suppresses Esr1 transcription and induces Esr1 degradation and that *Per2* is estrogen-inducible in Esr1 positive breast cancer cells (42). *Bmal1* knockdown down-regulates estrogen synthesis and aromatase expression in ovarian granulosa cells (43). Disrupted expression of circadian genes, including *Clock, Bmal1, Per2, Rev-erb*α, and *Cry1*, also occurs in Esr1 negative MCF10A mammary epithelial cells (44). We found that HFD altered diurnal oscillations of components of the female sex steroid hormone system. This includes elevated mesor of estrogen, Esr1 and Pgr and a reduced Esr2 mesor. Similarly, HFD increased concentrations of estrogen, Esr1 and Pgr and lowered that of Esr2 in mammary glands. Our findings support reports showing the existence of an interconnection between circadian signaling and steroid hormone signaling (39, 42) and indicate that a high fat diet may disrupt this interconnection.

Ovarian sex steroid hormones and their receptors play important roles in breast physiology (20, 21, 24). Mammary glands grow at the same rate as other organs until puberty, when elevations in ovarian hormones in blood circulation initiate morphological changes in mammary glands. Estrogen is essential for mammary development (20), but it is also a potent mitogen (45, 46). Thus, the elevated expression of estrogen, along with disrupted expression of Esr and Pgr, in pubertal mammary glands by a high fat diet may lead to aberrant mammary development. This is supported by a previous report that pubertal intake of a high fat diet alters mammary gland development in C57BL/6 mice (47).

In this study, we observed a lack of cyclical expression of Esr1 protein, compared to the cyclical expression of *Esr1*, in mammary glands from mice fed AIN93G diet. Moreover, we found that HFD delayed acrophase of *Pgr* but advanced acrophase of Pgr protein. These findings were unexpected. They suggest the possibility of post-translational modification or involvement of other mechanisms which may have affected the rhythmic expression during translation. This certainly warrants further investigation. Nevertheless, the elevated concentrations of Esr1 and Pgr proteins in mammary glands by HFD indicate the powerfulness of a high-fat consumption in modulating biological rhythms of pubertal mammary glands.

A limitation of this study is that we did not examine the pubertal stage of mice at the end of the study. This was to avoid circadian disruption by animal handling and vaginal lavage procedures. Furthermore, due to the limited quantity of mammary tissues available from 6-week old mice, additional experiments to evaluate proliferative and developmental status of mammary glands were not feasible. These measurements are certainly considerations for future studies, which can help better understand the roles of a high-fat diet on rhythmic alterations in pubertal mammary glands.

In summary, the present study showed the existence of diurnal oscillations of circadian genes, estrogen, progesterone, and their receptors in mammary glands of pubertal mice. Consumption of a HFD disrupted these oscillations and altered concentrations of estrogen, estrogen receptors, and progesterone receptor in mammary glands. These findings indicate that HFD-mediated circadian disruption may disturb the hormonal equilibrium of mammary glands, which may lead to aberrant development of mammary glands in pubertal mice.

## Data Availability Statement

The datasets generated for this study are available on request to the corresponding author.

## Ethics Statement

The animal study was reviewed and approved by IACUC Grand Forks Human Nutrition Research Center.

## Author Contributions

LY and SS designed the study, conducted experiments, collected data, interpreted results, and wrote the manuscript. LJ performed statistical analyses and contributed to manuscript writing. All authors contributed to review and revision of the manuscript and agreed to be accountable for the content of the work.

## Conflict of Interest

The authors declare that the research was conducted in the absence of any commercial or financial relationships that could be construed as a potential conflict of interest.

## Abbreviations

Bmal1: aryl hydrocarbon receptor nuclear translocator-like protein 1
Clock: circadian locomotor output cycles kaput
Cry.: Cryptochrome
Esr: estrogen receptor
HFD: high-fat diet
Per: period
Prg: progesterone receptor
Rev-erbα: nuclear receptor subfamily 1, group D, member 1.
